# In silico design of recombinant multi-epitope vaccine against influenza A virus

**DOI:** 10.1186/s12859-022-04581-6

**Published:** 2022-02-02

**Authors:** Avisa Maleki, Giulia Russo, Giuseppe Alessandro Parasiliti Palumbo, Francesco Pappalardo

**Affiliations:** 1 Department of Mathematics and Computer Science, University of Catania, 95125 Catania, Italy; 2 Department of Drug and Health Sciences, University of Catania, 95125 Catania, Italy

**Keywords:** Influenza A, Epitope prediction, Recombinant vaccine, Agent-based model

## Abstract

**Background:**

Influenza A virus is one of the leading causes of annual mortality. The emerging of novel escape variants of the influenza A virus is still a considerable challenge in the annual process of vaccine production. The evolution of vaccines ranks among the most critical successes in medicine and has eradicated numerous infectious diseases. Recently, multi-epitope vaccines, which are based on the selection of epitopes, have been increasingly investigated.

**Results:**

This study utilized an immunoinformatic approach to design a recombinant multi-epitope vaccine based on a highly conserved epitope of hemagglutinin, neuraminidase, and membrane matrix proteins with fewer changes or mutate over time. The potential B cells, cytotoxic T lymphocytes (CTL), and CD4 T cell epitopes were identified. The recombinant multi-epitope vaccine was designed using specific linkers and a proper adjuvant. Moreover, some bioinformatics online servers and datasets were used to evaluate the immunogenicity and chemical properties of selected epitopes. In addition, Universal Immune System Simulator (UISS) in silico trial computational framework was run after influenza exposure and recombinant multi-epitope vaccine administration, showing a good immune response in terms of immunoglobulins of class G (IgG), T Helper 1 cells (TH1), epithelial cells (EP) and interferon gamma (IFN-g) levels. Furthermore, after a reverse translation (i.e., convertion of amino acid sequence to nucleotide one) and codon optimization phase, the optimized sequence was placed between the two EcoRV/MscI restriction sites in the PET32a^+^ vector.

**Conclusions:**

The proposed “Recombinant multi-epitope vaccine” was predicted with unique and acceptable immunological properties. This recombinant multi-epitope vaccine can be successfully expressed in the prokaryotic system and accepted for immunogenicity studies against the influenza virus at the in silico level. The multi-epitope vaccine was then tested with the Universal Immune System Simulator (UISS) in silico trial platform. It revealed slight immune protection against the influenza virus, shedding the light that a multistep bioinformatics approach including molecular and cellular level is mandatory to avoid inappropriate vaccine efficacy predictions.

**Supplementary Information:**

The online version contains supplementary material available at 10.1186/s12859-022-04581-6.

## Background

Influenza has been for centuries a significant contributor to mortality and continues to be a significant threat to public health worldwide [1, 2]. The influenza virus belongs to the Orthomyxoviridae family and is divided into four subtypes: A, B, C, and D [3]. The influenza virus genome consists of several cRNA-segments which facilities viral variation by the mechanism of genetic reassortment [4]. The influenza A viruses have been responsible for causing the flu pandemic [5]. Influenza A virus structural proteins include hemagglutinin (HA) and neuraminidase (NA), which appear extensively on the lipid coating and serve the classify the virus. Currently, 18 HA and 11 NA subtypes are known, and 131 subtypes have been identified in nature [6]. HA protein can be divided into two functional domains, head and stem, encompassing highly conserved regions too; receptor-binding site (RBS) and the fusion peptide, respectively [7]. There are also two internal proteins: matrix protein (M1) and membrane matrix protein (M2). The M2 protein from the influenza A virus is crucial for infection. While the influenza A virus evolves rapidly with frequent mutation, the M2 protein, compared with other proteins encoded by the genome, comprises highly conserved residues [8]. These variations originate from two mutations: antigenic shift and antigenic drift, which allows the influenza virus to evade the human immune system [9]. Antigenic shift is caused by the substitution of hemagglutinin and sometimes neuraminidase through gene reassortment. New subtypes have not appeared in human viruses for a long time. Antigenic drift is caused by frequent point mutations during virus replication, affecting the antibody-binding sites in the HA protein, NA protein, or both.

Several vaccines have been developed for prophylaxis against human influenza viruses with the main target of HA. However, the function of these vaccines is limited due to the high mutation rate in the antigenicity of HA, short time for production, and the host's immune system. Consequently, vaccines are required to be frequently reformulated [10, 11]. Moreover, it is possible that sometimes the antigenicity of the vaccine does not match the epidemic viruses. One approach for improving the efficacy of vaccines is the approach of predicting the specific influenza A subtype that will be prevalent in a particular year. Prediction accuracy has decreased because of random genetic drift, incomplete samples of viruses that cause epidemics, and lack of knowledge regarding the evolution mechanism of sequences [12].

During the last decade, complex calculation techniques have been developed for predicting virus lineages, detecting genetic variations, and their functional impact. These techniques, such as in silico trials or thermostatted kinetic theory methods [13], ought also to be instrumental for vaccine design [14]. In silico trials use individual computer simulations to generate or evaluate a pharmaceutical product, medicinal equipment, or medical intervention. In the medical context they play a significant role in all aspects of diseases: prevention by designing and developing vaccines, diagnosis, prognostic appraisal, and prediction of the efficacy of specific treatment strategies [15]. In particular, considering the high mutation rate and evolutionary procedure in HA and NA, it is assumed that the conserved parts play a remarkable role in vaccine design [16]. In addition, the highly conserved M2 protein is valuable in the stability and improvement of vaccine function as it has 23 residues located outside the virus and assists M2 protein for the virion function [17, 18]. In this work, we evaluated the conserved parts of HA, NA, and M2, among the seven pathogenic strains, especially in Asia: H1N1, H1N2, H3N2, H5N1, H7N3, H7N9, and H9N2 by in silico method and combination as a single protein that can activate human humoral and cellular immunity [19–21].

The combination of epitope prediction tools and vaccine design methodologies alone do not frequently produce sufficient piece of evidence to evaluate the global immune response elicited by the vaccine under investigation. Agent based modeling can provide additional information useful to assess immune system elicited response at a cellular and organ level, closing the circle. For example, immune entities dynamics is revealed also in antigenic competition environment: this is not clearly predictable using only epitope prediction tools.

## Results

After applying this immunoinformatic procedure, related results of each step are reported below.

### Retrieving influenza protein sequences and multiple alignments

Amino acid sequences with FastA format for HA, NA, and M2 proteins strains were extracted from the NCBI database (Additional file 1). After multiple alignments by Jalview, consensus sequences for HA, NA, and M2 consist of 582, 257, and 487 amino acids, respectively.

### B-cell epitopes prediction

Epitopes with a length 10 to 20 were extracted from IEDB, and from SVMTriP only epitopes with a score above 0.5 were collected. Finally, 15 epitopes for HA, 11 epitopes for M2, and 12 epitopes for NA were chosen from these B-cell prediction tools.

### CTL epitopes prediction

15 supertype A2 ligand, 18 supertype A3 ligands, and 11 supertype B7 ligands were predicted for HA, M2, and NA proteins (Consensus peptide sequences) using NetCTL 1.2 server. Epitope identification threshold was set to 1; weight on C terminal cleavage, and TAP transport efficiency were set at to default.

### CD4 T cell epitopes prediction

A total of 40 strong bound epitopes without repetition were predicted using NetMHCIIpan–4.0 for human alleles HLA-DR, HLA-DQA1, and HLA-DQB1 (DRB1_1303, DRB1_1302, DRB1_1401, DRB1_0701, HLA-DQA10103-DQB10603, HLA-DQA10102-DQB10604, HLA-DQA10104-DQB10503, HLA-DQA10201-DQB10202, and HLA-DQA10201-DQB10303). NetMHCIIpan–4.0 web server was used based on their IC50 scores, and all parameters were set to default.

### Antigenicity and allergenicity prediction of CTL, CD4 T cell, and B cell epitopes

To select epitopes for the final recombinant vaccine, we evaluate the antigenicity, allergenicity, and toxicity of all 122 peptides (Additional file 2); then, we opted for non-allergenic and non-toxicity epitopes, which are antigens for the recombinant vaccine. Vaxigen provided antigenicity score for virus model is equal to 0.73 while AllerTOP 2.0 server predicted that the final recombinant vaccine is non-allergenic.

### Human population coverage analysis

Worldwide human population coverage analysis predicted that T-cell epitope based on the combination of HLA-I and HLA-II can cover 90.78% of the human population.

### Recombinant multi-epitope vaccine

The final vaccine, after considering some parameters for three adjuvants (PI, weight, half-life, etc.) has 813 amino acids and consists of a total of 40 epitopes including 11 CTL, 16 CD4 T cell, and 13 B cell peptides sequences (Table 1) (Additional file 3). The Adjuvant (A 50 S ribosomal protein L7/L12) was linked to N-terminal by EAAAK linker, and CTL, CD4 T cell, and B cell epitopes were merged using AYY, GPGPG, and KK linkers. AAY linkers significantly affect the expression of the target proteins and improve the immunogenicity of the multi-epitope vaccine. The significant feature of the GPGPG linker deals with its ability to break the junctional immunogenicity, which is caused by the amendment of the immunogenicity of each epitope, and GPGPG linkers have illustrated the ability to induce CD4 T cell responses which are essential for a multi-epitope vaccine. While the KK linker decreases the junctional immunogenicity by preventing the induction of antibodies for the peptide sequence that each epitope can form when joined linearly [22]. All linkers have pivotal roles in providing an extended conformation (flexibility), assisting folding, separating protein domains, and generally making the recombinant multi-epitope vaccine structure more stable [23]. Hence, from a general point of view, the possibility of introducing new "fake" epitopes in the linking regions would not represent a concrete issue to our best knowledge. A 6xHis tag was added to the C-terminal of the generated vaccine to increase protein purification and identification. The recombinant multi-epitope vaccine comprises several ectodomain locations, glycosylation sites, and solvent-accessible regions; while the selected B-cell epitopes shows averagely a score about 0.2 which mean the presence of suitable Relative Surface Accessibility regions (RSA).

**Table 1:** List of all the epitopes used in the construction of the recombinant multi-epitope vaccine

PROTEIN	CTL epitope	HTL epitope	B-CELL epitope
Hemagglutinin	KSYINNRGK	KPEIGARPKVNGQSG	TELLEDTHNGKLCDLKGVAPLDLG
	SSNYQQKFK	LIWLLKKNDNAAYPK	
	NIHPITIGK	SSLPFQNIHPITIGK	KGAINSSLPFQNIHP
	CPRAGSKSF	ANNSTTTVDTLTEKN	
	LPFQNIHPI	EQGSGYAADLKSTQK	LKLATGLRNV
	AVILAGLSF	LIEKMNTQFEAIDKE	
		NSTTTVDTLTEKNVE	
Neuraminidase	ITTVTLHFK	MGRTISEKSRSGYEM	TTLNNKHSNGTIHDRSP
		NQEIVNITNTIIEKE	GSASGQADTK
	ITGFAPFSK	NSKFQINRQDIVDID	YGTGSWPDGADINF
		NQEIVNITNTIIEKE	VRCVCRDNWKGSNRPWVDIN
Matrix	RMGTVNTEV	TNPLIRHENRMVLAS	PSGPTRNEWECRCS
		NPLIRHENRMVLAST	YGLKRVALSYST
	AVKLYRRFK	VLGFVFTLTVPSERG	RMGTVNTEVA
	GLKRVALSY	TEQQSAVDVDDGHFV	VQAMRTIGTEQQSAVDVDDG
		GTEQQSAVDVDDGHF	ELEQKRMGLQM
			GVLGFVFTLTVPSERGLQRR

Predicted linear B-cell epitopes and T-cell epitopes were selected from HA, NA, M2 proteins to design of the recombinant multi-epitope peptide

### Evaluation of physicochemical properties and solubility prediction

The molecular weight (MW) of the final vaccine is 87.3 KDa. The predicted theoretical pI is 9.35, and based on the pI of this protein is basic. The vaccine consists of 83 negatively charged residues and 108 positively charged residues. Half-life was estimated to be 30 h mammalian reticulocytes in vitro, > 20 h yeast in vivo, and > 10 h Escherichia coli in vivo. The formula is C3878H6146N1088O1171S18, and the total number of atoms is 12301. The Instability Index (II) is computed to be 27.74 and classifies the protein as stable. A protein with an instability index greater than 40 is unstable. The Aliphatic index was estimated to be 70.69, indicating thermostability. Furthermore, the last property is GRAVY which was predicted to be − 0.547. A negative GRAVY value indicates that the protein is non-polar and hydrophilic. The recombinant vaccine was evaluated as a soluble protein with a solubility score of 0.49.

### Secondary structure prediction of the recombinant vaccine

**Fig. 1:**
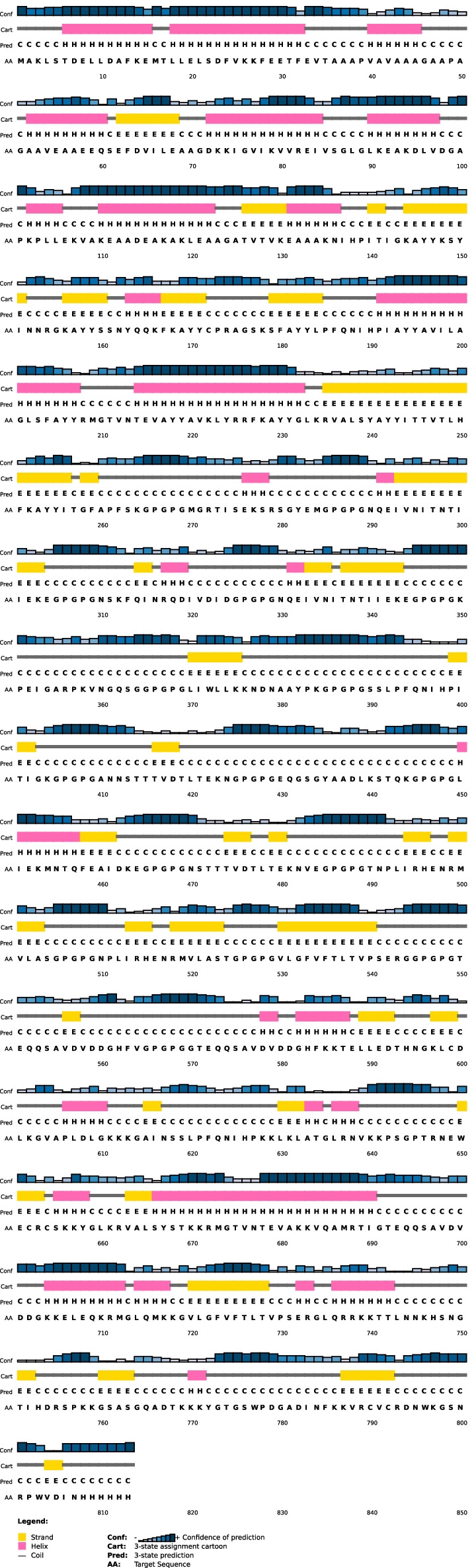
Prediction of secondary structure by PSIPRED. Graphical illustration of secondary structure features of the final recombinant multi-epitope vaccine sequence. The protein is estimated to contain alpha-helices (16%), beta strands (21%), and coils (61%)

According to the data obtained from PSIPRED, the final vaccine consists of 16% alpha-helix, 21% beta-sheet, and 61% coil, and 137 (16%) positions predicted as disordered. Predicting disordered regions is based on the cut-off value at 0.25 (Fig. 1). Another property is solvent accessibility, divided into three states by two cut-off values: 10% and 40%. This means that the three states have equal distribution: buried for less than 10%, exposed for larger than 40%, and medium for between 10 and 40%. Solvent accessibility was predicted to be 53% exposed, 24% medium exposed, and 22% buried.

### Codon adaption and in silico cloning of recombinant vaccine

**Fig. 2:**
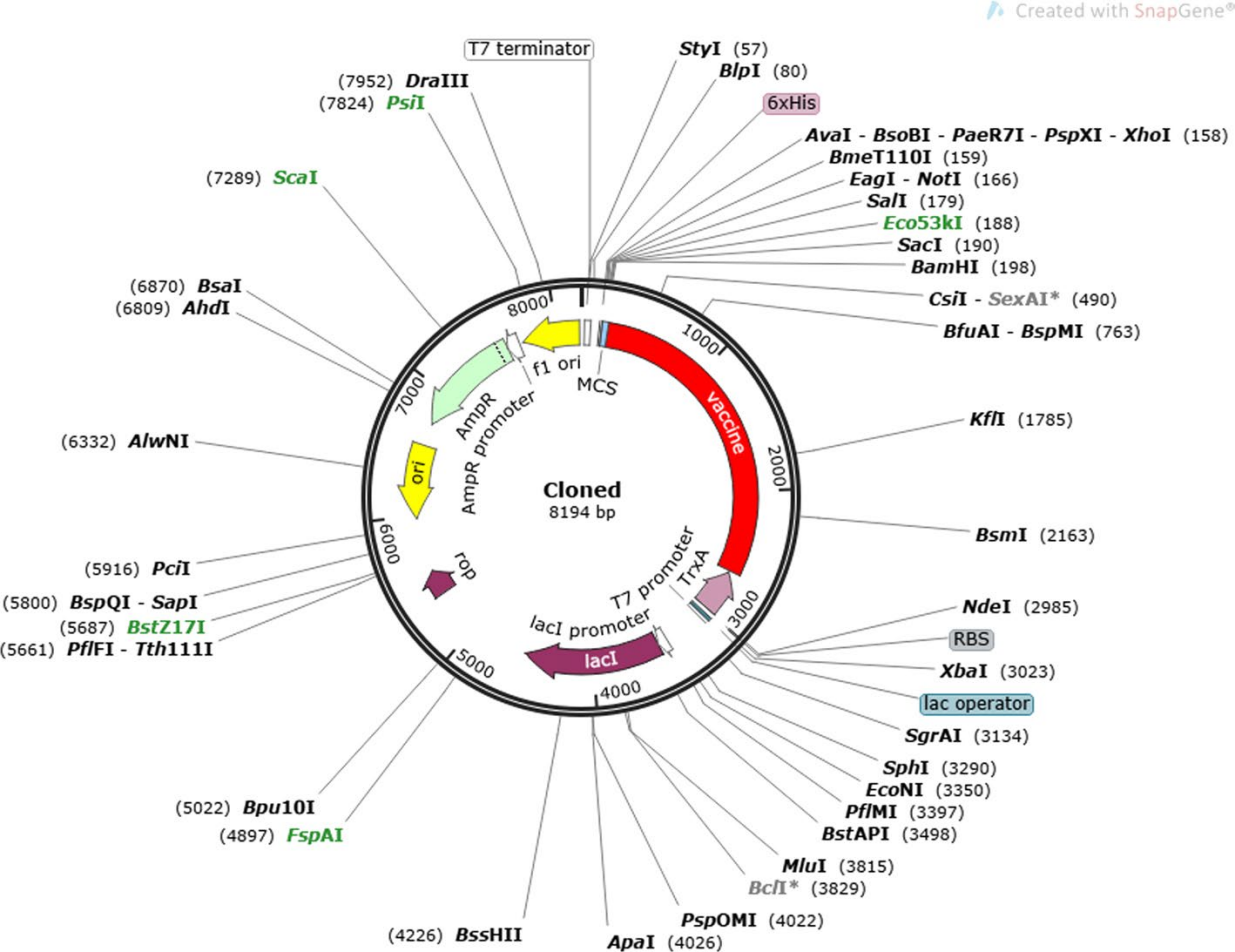
In silico cloning of vaccine candidate. In silico restriction cloning of the recombinant vaccine sequence (the red part) into the pET32a^+^ vector using EcoRV and MscI restriction site

JAVA Codon Adaptation tool was performed to optimize codon usage of the vaccine in E. coli (strain K12) for high protein expression. The optimized codon sequence length for a multi-epitope recombinant vaccine with 813aa was 2439 nucleotides. CAI value for optimized nucleotide sequence was 0.97, and CG-content of sequence was 50.88%, representing the excellent possibility expression of the recombinant vaccine in the E. coli host. SnapGene software was used to insert adapted codon sequences into pET32a^+^ vector by assisting EcoRV and MscI restriction enzymes. The final product (vector and optimized codon sequence) consists of 8194 bp (Fig. 2).

### In silico trial immune simulation

**Fig. 3:**
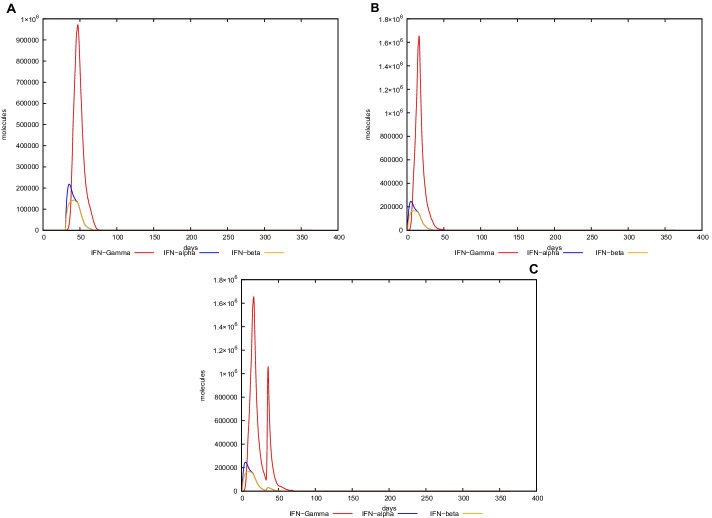
In silico dynamics of IFN-g through the UISS simulation platform. **A** IFN-g level after influenza exposure. **B** IFN-g level after the recombinant multi-epitope vaccine. **C** IFN-g levels after influenza exposure and recombinant multi-epitope vaccine administration

**Fig. 4:**
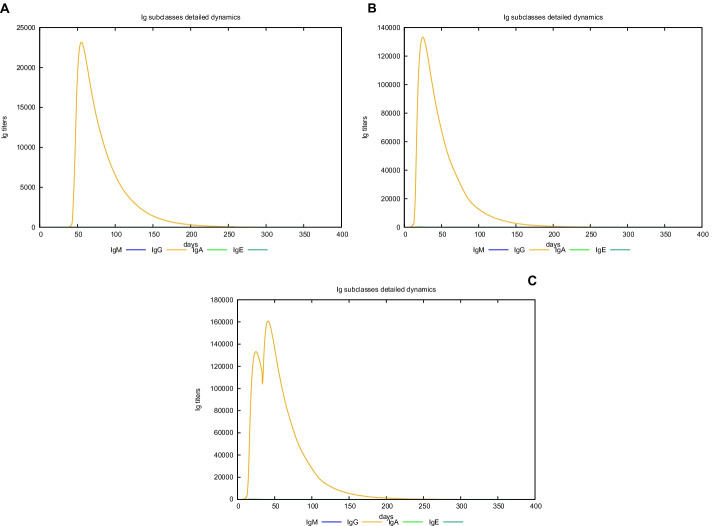
In silico dynamics of IgG through the UISS simulation platform. **A** IgG level after influenza exposure. **B** IgG level after the recombinant multi-epitope vaccine. **C** IgG levels after influenza exposure and recombinant multi-epitope vaccine administration

**Fig. 5:**
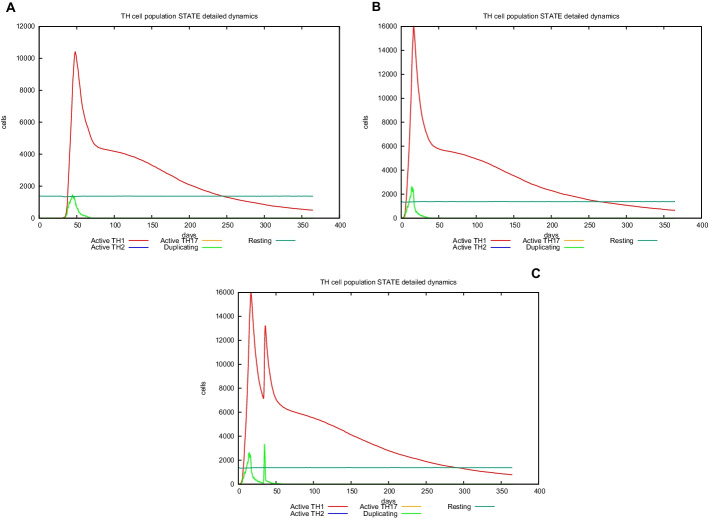
In silico dynamics of TH1 through the UISS simulation platform. **A** TH1 level after influenza exposure. **B** TH1 level after the recombinant multi-epitope vaccine. **C** TH1 levels after influenza exposure and recombinant multi-epitope vaccine administration

UISS computational platform was used to predict the immune simulation of the final recombinant multi-epitope vaccine. Here, we show in silico results of two specific scenarios in an average patient: (i) immune system dynamics after influenza exposure, (2) immune system dynamics after vaccine administration, and (3) immune system response to recombinant multi-epitope vaccine administration in presence of influenza exposure. In the first scenario, the peak level of IFN-g is about 1 × 10^6^ molecules at day 50 (Fig. 3, panel A), while in the second one, its level (about 1.6 × 10^6^ molecules is considerably higher than after influenza exposure at day 25 (Fig. 3, panel B). Figure 3, panel C shows a higher second peak as to highlight the effect of the vaccination in response to influenza challenge. Furthermore, the recombinant multi-epitope vaccine response is characterized by high levels of IgG, approximately 130,000 titers (Fig. 4, panel B), while after influenza exposure, IgG level is fewer (24,000 titers) compared to the one after vaccine simulation (Fig. 4, panels A–C). The recombinant multi-epitope vaccine responses demonstrate a notable increase in the number of TH1 cells (about 16,000 at day 30 (Fig. 5, panel B)). However, after influenza exposure, this amount is approximately 1000 cells at day 50 (Fig. 5, panel A). Figure 5, panel C, shows a higher second peak as to highlight the effect of the vaccination in response to influenza challenge.

**Fig. 6:**
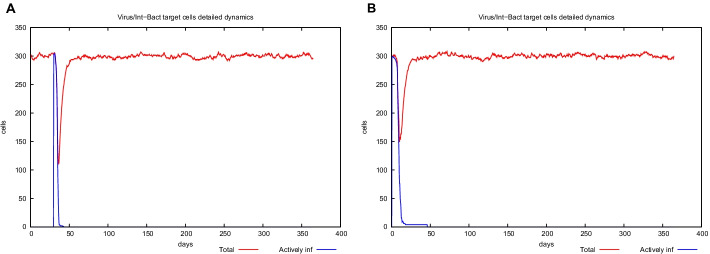
In silico dynamics of EP cells through the UISS simulation platform. **A** EP level after influenza exposure. **B** EP level after the recombinant multi-epitope vaccine

Still, after influenza exposure, the number of infected lung epithelial cells is slightly higher than in the vaccine administration scenario (Fig. 6, panels A-B). This means that the proposed multi-epitope vaccine could elicit an immune response that partially protects from the infection.

## Discussion

Influenza is one of the most significant contagious respiratory infection diseases, and despite vaccination, it is still one of the leading causes of mortality and threatens worldwide public health [24]. The generation of new multi-epitope vaccines brings various advantages in comparison to other approaches. Infectious substances or perilous sequences can be extracted, thus reducing the risk of undesired host reactions. Furthermore, multi-epitope vaccines are not at risk of relapse, because are weak or live vaccines [25]. Also, from a pharmaceutical point of view, multi-epitope vaccines demonstrate some desirable properties. Because multi-epitope vaccines are based on chemically well-characterized peptides, they can be produced efficiently and cost-effectively. The multiple-epitope vaccine can cover a wide range of pathogens or strains of a particular pathogen, especially for highly variable pathogens such as influenza virus, which faces several mutations and generates novel variants [26].

Animal studies demonstrate that T lymphocytes can induce a protective immune response against the influenza virus by identifying proteins processed and delivered by MHC molecules. CTL can detect several epitopes in the HA structure. Due to this fact, the response of CTL to epitope vaccines is entirely dependent on the structure of the HLA molecule. Therefore, in designing multi-epitope vaccines, T lymphocyte epitopes should be selected according to their power to elicit a response in the most of the population [27, 28]. In addition to T lymphocytes, the importance of CD4 + cells has also been considered during the immune response to the influenza virus [29]. By identifying the peptides provided by MHCII molecules, they initiate and amplify the dependent responses of CD8 + and B lymphocytes against influenza virus infection [30]. Conserved regions in HA, NA, and M are the main target to design recombinant protein as a multi-epitope vaccine which can be presented by both MHCI and MHCII and activates cellular or humoral responses.

A trial platform such as UISS computational framework is helpful in evaluating the goodness of vaccine efficacy designed through available bioinformatics tools, enhancing their success probability when tested in pre-clinical and clinical settings. However, a multi-epitope vaccine has some limitations; for instance, one of the significant limitations of a multi-epitope vaccine that most epitope prediction tools do not suitably consider is the need to distinguish proper antigen processing sites that can lead to the prediction and presentation of predicted epitopes. Because the composition of antigen processing mechanisms varies based on proinflammatory signals and can vary among different cell classes, currently existing prediction algorithms may not be proper to evaluating the processing effectiveness of viral antigens in an infected target cell [31].

Here, we evaluated HA, NA, and M2 proteins in pathogenic strains in Asia (H1N1, H1N2, H3N2, H5N1, H7N3, H7N9, and H9N2). Consensus sequences for each protein were identified after extracting and blasting sequences of HA, NA, and M2 proteins for seven pathogenic strains. Consensus sequences comprise highly conserved residues. Then, B-cell linear, CTL, and CD4 T cell epitopes were predicted, and epitopes with high scoring and high affinity were selected for calculating antigenicity, allergenicity, and toxicity for the individual peptides, as well as for the entire vaccine. Vaxijen v2.0 default threshold for showing antigenicity is equal to 0.4; therefore, epitopes with scores above 0.4, non-toxic, and non-allergenic, were chosen for designing a recombinant vaccine. To select the suitable adjuvant, three peptides were evaluated: a 50 S ribosomal protein L7/L12, H9E, and MDA5. L7/L12 seems to be a more appropriate choice. The past study reported that AAY, GPGPG, and EAAAK linkers were used between the predicated epitopes to generate a sequence with minimized junctional immunogenicity, allowing the rational design of a potent recombinant multi-epitope vaccine. Codon optimization was carried out to achieve high-level expression of the recombinant multi-epitope vaccine in the 12 K strain of E. coli. CAI value for optimized nucleotide sequence was 0.97, and CG-content was equal to 50.88%, showing the excellent possibility of expression of the multi-epitope vaccine.

## Conclusions

This study deals with the design of a recombinant vaccine against influenza A, especially against seven pandemic strains in Asia (H1N1, H1N2, H3N2, H5N1, H7N3, H7N9, and H9N2), based on conserved residues of HA, NA, and M2 proteins. B cell linear, CTL, and CD4 T cell epitopes were predicted using online servers, and after spreading high scoring and high-affinity epitopes, antigen, non-allergic and non-toxic epitopes were selected for the recombinant vaccine. Epitopes were linked together by several different linkers to reduce junctional immunogenicity. Population coverage was calculated, and this recombinant vaccine can cover 90.78% of the worldwide population. Then, codon optimization was carried out for cloning and expression of the vaccine in *E. coli* (strain K12). CIA and CG-content indicate a high level of expression in E. coli. Then, the recombinant vaccine was inserted into the pET32a^+^ vector by assisting EcoRV and MscI restriction enzyme for cloning. The resulting suggested vaccine formulation was found with a high immunogenicity score. However, further investigations conducted with UISS in silico platform highlighted a partial immune system protection response elicited by the designed multi-epitope vaccine formulation. A multistep bioinformatic approach would hence ameliorate the vaccine development pipeline enhancing the probability of keeping good results in pre-clinical and clinical settings. The recombinant multi-epitope vaccine is an entirely hypothetical protein construct with no experimental verified epitopes; therefore, we can claim that all positive results obtained belong to the in silico level. Further experimental studies, along with epitope confirmation, should be performed.

## Methods

**Fig. 7:**
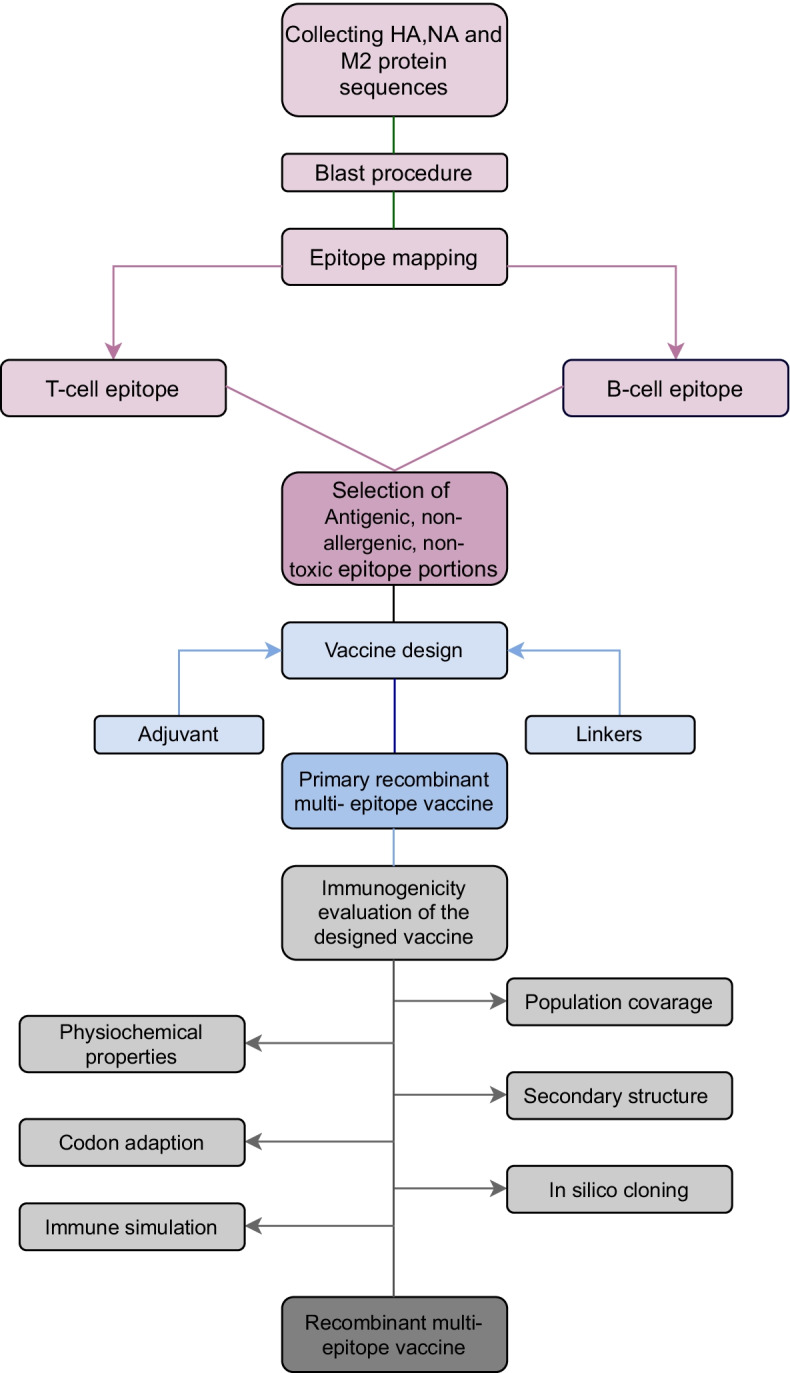
Workflow of the multi-bioinformatic approach. Graphical illustration of the step-by-step phases used for the in silico design of the proposed recombinant multi-epitope vaccine against influenza A virus

In this section, the specific steps involved in designing the recombinant multi-epitope vaccine against influenza are reported in detail through specific subparagraphs. In parallel, a sketch of the entire workflow of the multi-bioinformatic workflow is depicted in Fig. 7.

The online services have been all accessed on August, 10th 2021.

### Retrieving influenza protein sequences and multiple alignments

The amino acid sequences of HA, NA, and M2 proteins for seven strains (H1N1, H1N2, H3N2, H5N1, H7N3, H7N9, and H9N2) have been revealed from the NCBI database [32]. These seven strains include chicken, swine, and goose sequences to cover a wide range of influenza viruses. Separately, multiple alignments were performed by Jalview software based on the Muscle algorithm for seven strains of HA, seven strains of NA, and seven strains of M2 to identify consensus sequences for each protein [33] (Additional file 1).

### B-cell epitopes prediction

The main purpose of predicting B-cell epitopes is to develop synthetic peptide vaccines, in which case the predicted epitopes must also be able to elicit antibodies that neutralize the infectivity pathogen harboring the protein antigen [34]. Therefore, there are various databases to predict B-cell epitopes. This study used SVMTriP (http://sysbio.unl.edu/SVMTriP/prediction.php) and IEDB Analysis (http://tools.iedb.org/bcell/) resources to predict B-cell linear epitopes. In this method, a support vector machine (SVM) with a combination of three peptide similarities and propensity scores (SVMTriP) is used to achieve better predictive performance [35]. To use SVMTriP the epitope length was set to 20 amino acids. Bepipred Linear Epitope Prediction 2.0 method was chosen to use IEDB Analysis tools and the residues with scores above the threshold (default value is 0.5) are predicted to be part of an epitope. The sensitivity of 0.5 threshold is 0.58564 and the specificity is 0.57158, which are superior to other available tools for sequence-based epitope prediction [36].

### CTL epitopes prediction

MHC class I epitopes were identified by NetCTL 1.2 server (http://www.cbs.dtu.dk/services/NetCTL/) for three selected proteins. The method integrates peptide MHC class I binding prediction, proteasomal C terminal cleavage, and Transporter Associated with Antigen Processing (TAP) transport efficiency. The server provides predictions of CTL epitopes restricted to 12 MHC class I supertypes. MHC class I binding and proteasomal cleavage are performed using artificial neural networks. TAP transport efficiency is predicted using a weight matrix [37]. Recent studies on the influenza virus have shown that the epitopes of HLA class I (-A2, -A3, or -B7 supertypes) are highly conserved among different influenza virus strains. Therefore, they have a high potential for the immunity of the CTL-based vaccine against all serotypes of the influenza virus [38]. In this study, the threshold value for epitope identification was set to 1; weight on C terminal cleavage was set to 0.15, and weight on TAP transport efficiency was set to 0.05 to predict CTL epitopes.

### CD4 T cell epitopes prediction

NetMHCIIpan–4.0 (https://services.healthtech.dtu.dk/service.php?NetMHCIIpan-4.0) was used to predict MHC class 2 epitopes with a length of 15- Mer for human alleles and threshold for strong binder (% Rank) was set to 2, while threshold for weak binder (% Rank) was set to 10. Both of them are default settings. NetMHCIIpan predicts epitope binding to any MHC II molecule of known sequence and covers the three human HLA-DR, HLA-DQ, and HLA-DP alleles using artificial neural networks (ANNs). NetMHCIIpan has been reported to predict T cell epitopes with great accuracy [39]. Based on extensive research, it has been confirmed that DRB1_1303, DRB1_1302, DRB1_1401, DRB1_0701, HLA-DQA10103-DQB10603, HLA-DQA10102-DQB10604, HLA-DQA10104-DQB10503, HLA-DQA10201-DQB10202, and HLA-DQA10201-DQB10303 are frequent in all populations; therefore, they have been selected for the present study [40]. All parameters were set to the default value, and only strong binding peptides were included in this study.

### Antigenicity and allergenicity prediction of CTL, CD4 T cell, and B-cell epitopes

Antigenicity, allergenicity, and toxicity were predicted for each CTL, CD4 T cell, and B-Cell epitopes. VaxiJen v2.0 (http://www.ddg-pharmfac.net/vaxijen/VaxiJen/VaxiJen.html) was applied to determine the antigenicity of the peptidesand AllerTOP v2.0 (https://www.ddg-pharmfac.net/AllerTOP/) to evaluate allergenicity of the peptides, while ToxinPred (https://webs.iiitd.edu.in/raghava/toxinpred/design.php) was used to show the toxicity of the peptides [41–43]. The prediction method of ToxinPred was set to SVM (Swiss-Prot) + Motif based and E-value cut-off for motif-based method was set to 10. Physicochemical properties option was set to “all”. The method of VaxiJen is based on the physicochemical properties of proteins without recourse to sequence alignment. The threshold for VaxiJen was set to 0.4 (default), and the target organism selected was the virus.

### Human population coverage analysis

The vaccines that are being designed should cover a wide range of the world population. Also, the extension of Human Leukocyte Antigens (HLA) diversity varies in different populations [44]. To determine worldwide human population coverage, IEDB (http://tools.iedb.org/population/) was used to evaluate 16 HLA class I and class II alleles considered in this study [44]. The area option was set to “world” and calculation option was set to “Class I and II combined”.

### Recombinant multi-epitope vaccine

We selected antigen, non-allergenic, and non-toxicity epitopes from high-scoring CTL, high-affinity CD4 T cell, and B-cell epitopes with scoring above 0.5 to generate a multi-epitope vaccine. Three peptide adjuvants were chosen for further analysis; A 50 S ribosomal protein L7/L12 (accession no. P9WHE3), H9E, and MDA5. After checking some parameters, the candidate adjuvant was selected for the final vaccine [45–47]. Adjuvants have a pivotal role in increasing the immunogenicity of the vaccine. For joining adjuvant to the N-terminal, EAAAK linker was used. EAAAK is a stable and rigid α-helical peptide linker that includes an intramolecular hydrogen bond and a closed-packed backbone. Therefore, the EAAAK linker has a domain spacer's role in a fusion protein [48]. To merge CTL, CD4 T cell and B-cell epitopes, AYY, GPGPG, and KK linkers were used, respectively, and also a 6xHis tag was added at the C-terminal part to improve protein purification and identification [49]. Ectodomain location, glycosylation sites, and solvent-accessible regions were predicted for the multi-epitope vaccine using the BCEPS web server (http://imbio.med.ucm.es/bceps/)and NetSurfP (https://services.healthtech.dtu.dk/service.php?NetSurfP-1.1) used to evaluate solvent accessible regions for selected B-cell epitopes. The NetSurfP server measures the solvent accessible regions of all amino acids in each selected B-cell epitope [50]. The parameters for BCEPS web server were set to default, which means the model was set to SVM; the number of aa was set at 16; the threshold was set to 0.5 and the immunogenicity was set for considering both CD4 and any human. The recombinant multi-epitope vaccine is the synthetic protein at this level.

### Evaluation of physicochemical properties and solubility

ProtParam (https://web.expasy.org/protparam/) was used to indicate various physicochemical properties of the recombinant vaccine included the number of amino acids, molecular weight, theoretical isoelectric point (pI), amino acid composition, atomic composition, chemical formula, extinction coefficients, estimated half-life, instability index, aliphatic index, and grand average of hydropathicity (GRAVY) [51]. The solubility of the recombinant vaccine was evaluated using the Protein-sol server (https://protein-sol.manchester.ac.uk). The population average for the experimental dataset (PopAvrSol) is 0.45. Therefore, any scaled solubility value greater than 0.45 is predicted to have a higher solubility than the average soluble E. coli protein from the experimental solubility dataset. Moreover, any protein with a lower scaled solubility value is predicted to be less soluble [52].

### Secondary structure prediction of the recombinant vaccine

PSIPRED 4.0 web server (http://bioinf.cs.ucl.ac.uk/psipred/) was used to predict the secondary structure of the final vaccine formulation [53]. In this study, amino acid sequences were used as an input to predict the secondary structure. Secondary structure properties were predicted using the RaptorX Property web server (http://raptorx.uchicago.edu/StructurePropertyPred/predict/) [54–56].

### Codon adaption and in silico cloning of the recombinant vaccine

After selecting the most suitable vaccine candidate based on bioinformatics analysis, JAVA Codon Adaptation Tool (JCat) (http://www.jcat.de/Start.jsp) was utilized for reverse translation and codon optimization for vaccine candidates to express in the *E. coli* (strain K12) host. Codon optimization is a technique that significantly increases gene expression in the expression vector and host cell. All parameters of the additional options section, such as avoid rho-independent transcription terminators, prokaryotic ribosome binding sites, and cleavage sites of restriction enzymes, were selected. The output of Jcat consists of the codon adaption index (CAI-Value) and CG-content of the improved sequence. The ideal score for the CAI index is 1.0, but > 0.8 is considered a great score, and for CG- content is among 30–70% [57]. By performing the SnapGene tool, the *E. coli* pET-32a^+^ vector was used to clone the optimized nucleotide sequence of the final recombinant vaccine construct. The pET system is one of the advanced systems to clone and express recombinant proteins such as multiple-epitope vaccine in *E. coli.* One disadvantage of using a pET system is that, despite adjustment by the lac repressor, it can still sometimes leak slightly (up to 5% in some cases). Thus, this may not be the best option if the protein under examination has significant effects in small amounts. Also, very hydrophobic proteins can produce some toxic, so those should be avoided when applied in this system. On the other hand, the pET-32a + plasmid owns a high bacterial expression, and it can produce soluble, active target proteins [58]. Then, EcoRV and MscI restriction sites were introduced to the N and C-terminals of the sequence, respectively.

### In silico trial immune simulation

To further evaluate the immunogenicity and related immune response profile of the recombinant protein, an agent-based methodology through Universal Immune System Simulator (UISS) was applied. UISS is an agent-based model (ABM) [59] developed firstly for tumor immunology and then adapted, through different stages of immune system features development, to comprehensive disease modeling scenarios including influenza and other infectious diseases [60–63]. This model can reproduce and capture the immune system dynamic both from a humoral and cellular point of view [64].

In this specific case study, the simulations we run represent the mean patient for three different scenarios: immune system dynamics after H1N1 influenza strain exposure, immune system dynamics after vaccine administration, and immune system dynamics after influenza exposure and vaccine administration. The time step for the first scenario is set at 90, while the second one is set at 2; the replication rate is equal to 60.0 h.

## Supplementary Information

**Additional file 1**: Nucleotide and protein gene bank ID of HA, NA, and M2 proteins.

**Additional file 2**: All linear B-cell, CTL, and HTL epitopes with antigenicity index.

**Additional file 3**: Comparison adjuvants properties.

## Data Availability

Datasets used in the experiments are listed as follows: (1) NCBI: National Center for Biotechnology Information (nih.gov). (2) SVMTriP: http://sysbio.unl.edu/SVMTriP/prediction.php. (3) IEDB: http://tools.iedb.org/bcell/. (4) NetCTL 1.2 server: http://www.cbs.dtu.dk/services/NetCTL/. (5) NetMHCIIpan–4.0: https://services.healthtech.dtu.dk/service.php?NetMHCIIpan-4.0. (6) VaxiJen v2.0: http://www.ddg-pharmfac.net/vaxijen/VaxiJen/VaxiJen.htmlt. (7) AllerTOP v2.0: https://www.ddg-pharmfac.net/AllerTOP/. (8) ToxinPred: https://webs.iiitd.edu.in/raghava/toxinpred/design.php. (9) IEDB: http://tools.iedb.org/population/. (10) BCEPS web server: http://imbio.med.ucm.es/bceps/. (11) NetSurfP: https://services.healthtech.dtu.dk/service.php?NetSurfP-1.1. (12) ProtParam: https://web.expasy.org/protparam/. (13) Protein-sol server: https://protein-sol.manchester.ac.uk. (14) PSIPRED 4.0 web server: http://bioinf.cs.ucl.ac.uk/psipred/. (15) RaptorX Property web server: http://raptorx.uchicago.edu/StructurePropertyPred/predict/. (16) JAVA Codon Adaptation Tool (JCat): http://www.jcat.de/Start.js

## Abbreviations

IgG: Immunoglobulins of class G
TH1: T Helper 1 cells
EP: Epithelial cells
IFN-g: Interferon gamma
HA: Hemagglutinin
NA: Neuraminidase
RBS: Receptor-binding site
M1: Matrix protein
M2: Membrane matrix protein
SVM: Support vector machine
TAP: Transporter associated with antigen processing
ANNs: Artificial neural networks
HLA: Human leukocyte antigens
pI: Isoelectric point
GRAVY: Grand average of hydropathicity
JCat: JAVA codon adaptation tool
CAI: Codon adaption index
UISS: Universal Immune System Simulator
ABM: Agent based model
RSA: Relative Surface Accessibility regions
MW: Molecular weight
II: Instability index

## References

[1] Kilbourne ED (2006). Influenza pandemics of the 20th century. Emerg Infect Dis.

[2] Guo C, Xie X, Li H, Zhao P, Zhao X, Sun J (2015). Prediction of common epitopes on hemagglutinin of the influenza A virus (H1 subtype). Exp Mol Pathol.

[3] Hause BM, Collin EA, Liu R, Huang B, Sheng Z, Lu W, et al. Characterization of a novel influenza virus in cattle and swine: proposal for a new genus in the Orthomyxoviridae family. mBio. 2014;5. 10.1128/mBio.00031-14 PMC3958797 24595369

[4] Widjaja I, de Vries E, Rottier PJM, de Haan CAM. Competition between influenza A virus genome segments. PLoS ONE. 2012;7. 10.1371/journal.pone.0047529 PMC3469491 23071819

[5] Bouvier NM, Palese P (2008). The biology of influenza viruses. Vaccine.

[6] Tong S, Zhu X, Li Y, Shi M, Zhang J, Bourgeois M, et al. New world bats harbor diverse influenza A viruses. PLOS Pathogens. 2013;9:e1003657. 10.1371/journal.ppat.1003657 PMC3794996 24130481

[7] Wu NC, Wilson IA. Structural insights into the design of novel anti-influenza therapies. Nat Struct Mol Biol. 2018;25:2. 2018;25:115–21. 10.1038/s41594-018-0025-9 PMC5930012 29396418

[8] Guo Y, He L, Song N, Li P, Sun S, Zhao G (2017). Highly conserved M2e and hemagglutinin epitope-based recombinant proteins induce protection against influenza virus infection. Microbes Infect.

[9] K N. [The mechanism of antigenic shift and drift of human influenza virus]. Nihon rinsho Jpn J Clin Med. 2003;61:1897–903. 14619428

[10] Bedford T, Riley S, Barr IG, Broor S, Chadha M, Cox NJ, et al. Global circulation patterns of seasonal influenza viruses vary with antigenic drift. Nature 2015 523:7559. 2015;523:217–20. 10.1038/nature14460 PMC4499780 26053121

[11] Carrat F, Flahault A (2007). Influenza vaccine: the challenge of antigenic drift. Vaccine.

[12] KimHyunsuh, G. W, J. W. Influenza virus: dealing with a drifting and shifting pathogen. https://home.liebertpub.com/vim. 2018;31:174–83. 10.1089/vim.2017.0141 29373086

[13] Bianca C, Riposo J, Bianca C, Riposo J (2015). Mimic therapeutic actions against keloid by thermostatted kinetic theory methods. EPJP.

[14] Kanyiri CW, Mark K, Luboobi L. Mathematical analysis of influenza A dynamics in the emergence of drug resistance. Comput Math Methods Med. 2018;2018. 10.1155/2018/2434560 PMC6136569 30245737

[15] Viceconti M, Henney A, Morley-Fletcher E (2016). In silico clinical trials: how computer simulation will transform the biomedical industry. Int J Clin Trials.

[16] Palese P, García-Sastre A (2002). Influenza vaccines: present and future. J Clin Investig.

[17] Farahmand B, Taheri N, Shokouhi H, Soleimanjahi H, Fotouhi F (2019). Chimeric protein consisting of 3M2e and HSP as a universal influenza vaccine candidate: from in silico analysis to preliminary evaluation. Virus Genes.

[18] Ponomarenko J, Bui H-H, Li W, Fusseder N, Bourne PE, Sette A, et al. ElliPro: a new structure-based tool for the prediction of antibody epitopes. BMC Bioinform. 2008;9:1–8. 10.1186/1471-2105-9-514 PMC2607291 19055730

[19] Hampson A, Barr I, Cox N, Donis RO, Siddhivinayak H, Jernigan D (2017). Improving the selection and development of influenza vaccine viruses—report of a WHO informal consultation on improving influenza vaccine virus selection, Hong Kong SAR, China, 18–20 November 2015. Vaccine.

[20] Nili H, Asasi K (2003). Avian influenza (H9N2) outbreak in Iran. Avian Dis.

[21] Kim JY (2016). The 2009 H1N1 pandemic influenza in Korea. Tubercul Respir Dis.

[22] Sajjad R, Ahmad S, Azam SS. In silico screening of antigenic B-cell derived T-cell epitopes and designing of a multi-epitope peptide vaccine for Acinetobacter nosocomialis. J Mol Graph Model. 2020;94:107477. 10.1016/j.jmgm.2019.107477 31654980

[23] Chen X, Zaro JL, Shen WC (2013). Fusion protein linkers: property, design and functionality. Adv Drug Deliv Rev.

[24] Steinbruck L, Klingen TR, McHardy AC (2014). Computational prediction of vaccine strains for human influenza A (H3N2) viruses. J Virol.

[25] Schubert B, Lund O, Nielsen M. Evaluation of peptide selection approaches for epitope-based vaccine design. 2013;82:243–51. 10.1111/tan.12199 24461003

[26] Purcell AW, McCluskey J, Rossjohn J (2007). More than one reason to rethink the use of peptides in vaccine design. Nat Rev Drug Discov.

[27] Sheikh QM, Gatherer D, Reche PA, Flower DR (2016). Towards the knowledge-based design of universal influenza epitope ensemble vaccines. Bioinformatics.

[28] Sun Y, Shi Y, Zhang W, Li Q, Liu D, Vavricka C, et al. In silico characterization of the functional and structural modules of the hemagglutinin protein from the swine-origin influenza virus A (H1N1)-2009. Sci China Life Sci. 2010 53:6. 2010;53:633–42. 10.1007/s11427-010-4010-8 20602265

[29] Altenburg AF, Rimmelzwaan GF, de Vries RD (2015). Virus-specific T cells as correlate of (cross-)protective immunity against influenza. Vaccine.

[30] Durães-Carvalho R, Salemi M (2018). In-depth phylodynamics, evolutionary analysis and in silico predictions of universal epitopes of Influenza A subtypes and Influenza B viruses. Mol Phylogenet Evol.

[31] Silva-Arrieta S, Goulder PJR, Brander C. In silico veritas? Potential limitations for SARS-CoV-2 vaccine development based on T-cell epitope prediction. PLOS Pathogens. 2020;16:e1008607. 10.1371/journal.ppat.1008607 PMC7272002 32497149

[32] Pruitt KD, Tatusova T, Maglott DR. NCBI Reference Sequence (RefSeq): a curated non-redundant sequence database of genomes, transcripts and proteins. Nucl Acids Res. 2005;33 suppl_1:D501–4. 10.1093/nar/gki025 PMC539979 15608248

[33] Waterhouse AM, Procter JB, Martin DMA, Clamp M, Barton GJ (2009). Jalview Version 2—a multiple sequence alignment editor and analysis workbench. Bioinformatics.

[34] Potocnakova L, Bhide M, Pulzova LB. An introduction to B-cell epitope mapping and in silico epitope prediction. J Immunol Res. 2016;2016. 10.1155/2016/6760830 PMC5227168 28127568

[35] Yao B, Zhang L, Liang S, Zhang C. SVMTriP: a method to predict antigenic epitopes using support vector machine to integrate tri-peptide similarity and propensity. PLOS ONE. 2012;7:e45152. 10.1371/journal.pone.0045152 PMC3440317 22984622

[36] Jespersen MC, Peters B, Nielsen M, Marcatili P. BepiPred-2.0: improving sequence-based B-cell epitope prediction using conformational epitopes. Nucl Acids Res. 2017;45:W24–9. 10.1093/nar/gkx346 PMC5570230 28472356

[37] Peters B, Bulik S, Tampe R, van Endert PM, Holzhütter H-G (2003). Identifying MHC class I epitopes by predicting the TAP transport efficiency of epitope precursors. J Immunol.

[38] Staneková Z, Varečková E (2010). Conserved epitopes of influenza A virus inducing protective immunity and their prospects for universal vaccine development. Virol J..

[39] Nielsen M, Lund O. NN-align. An artificial neural network-based alignment algorithm for MHC class II peptide binding prediction. BMC Bioinform. 2009;10:1–10. 10.1186/1471-2105-10-296 PMC2753847 19765293

[40] Reveille JD, Bruce GS. MHC Class II and non-MHC genes in the pathogenesis of systemic lupus erythematosus. Systemic Lupus Erythematosus: Fourth Edition. 2004; pp. 109–51.

[41] Doytchinova IA, Flower DR. VaxiJen: a server for prediction of protective antigens, tumour antigens and subunit vaccines. BMC Bioinform. 2007;8:1–7. 10.1186/1471-2105-8-4 PMC1780059 17207271

[42] Dimitrov I, Bangov I, Flower DR, Doytchinova I. AllerTOP v.2—a server for in silico prediction of allergens. J Mol Model. 2014;20:1–6. 10.1007/s00894-014-2278-5 24878803

[43] Gupta S, Kapoor P, Chaudhary K, Gautam A, Kumar R, Consortium OSDD, et al. In silico approach for predicting toxicity of peptides and proteins. PLoS ONE. 2013;8:e73957. 10.1371/journal.pone.0073957 PMC3772798 24058508

[44] Bui H-H, Sidney J, Dinh K, Southwood S, Newman MJ, Sette A. Predicting population coverage of T-cell epitope-based diagnostics and vaccines. BMC Bioinform. 2006;7:1–5. 10.1186/1471-2105-7-153 PMC1513259 16545123

[45] Liniger M, Summerfield A, Ruggli N. MDA5 can be exploited as efficacious genetic adjuvant for DNA vaccination against lethal H5N1 influenza virus infection in chickens. PLOS ONE. 2012;7:e49952. 10.1371/journal.pone.0049952 PMC3515599 23227156

[46] Hongzhou H, Jishu S, Julia L, Ziyan L, McVey DS, Sun XS. Design of a shear-thinning recoverable peptide hydrogel from native sequences and application for influenza H1N1 vaccine adjuvant. Soft Matter. 2011;7:8905–12.

[47] Khatoon N, Pandey RK, Prajapati VK (2017). Exploring Leishmania secretory proteins to design B and T cell multi-epitope subunit vaccine using immunoinformatics approach. Sci Rep.

[48] Dong R, Chu Z, Yu F, Zha Y. Contriving multi-epitope subunit of vaccine for COVID-19: immunoinformatics approaches. Front Immunol. 2020;0:1784. 10.3389/fimmu.2020.01784 PMC7399176 32849643

[49] Ayyagari VS, C. VT, K. AP, Srirama K. Design of a multi-epitope-based vaccine targeting M-protein of SARS-CoV2: an immunoinformatics approach (2020). 10.1080/07391102.2020.1850357 10.1080/07391102.2020.1850357 PMC7754933 33252008

[50] Ras-Carmona A, Pelaez-Prestel HF, Lafuente EM, Reche PA (2021). BCEPS: a web server to predict linear B cell epitopes with enhanced immunogenicity and cross-reactivity. Cells.

[51] Gasteiger E, Hoogland C, Gattiker A, Duvaud S, Wilkins MR, Appel RD, et al. Protein identification and analysis tools on the ExPASy server. The proteomics protocols handbook. 2005;, pp 571–607.

[52] Hebditch M, Carballo-Amador MA, Charonis S, Curtis R, Warwicker J (2017). Protein–Sol: a web tool for predicting protein solubility from sequence. Bioinformatics.

[53] McGuffin LJ, Bryson K, Jones DT (2000). The PSIPRED protein structure prediction server. Bioinformatics.

[54] Wang S, Peng J, Ma J, Xu J (2016). Protein secondary structure prediction using deep convolutional neural fields. Sci Rep.

[55] Wang S, Li W, Liu S, Xu J (2016). RaptorX-property: a web server for protein structure property prediction. Nucl Acids Res.

[56] Yang Y, Gao J, Wang J, Heffernan R, Hanson J, Paliwal K (2018). Sixty-five years of the long march in protein secondary structure prediction: the final stretch?. Brief Bioinform.

[57] Grote A, Hiller K, Scheer M, Münch R, Nörtemann B, Hempel DC, et al. JCat: a novel tool to adapt codon usage of a target gene to its potential expression host. Nucl Acids Res. 2005;33 suppl_2:W526–31. 10.1093/nar/gki376 PMC1160137 15980527

[58] pET Bacterial Recombinant Protein Expression Vector | VectorBuilder. https://en.vectorbuilder.com/resources/vector-system/pET16b.html. Accessed 29 Oct 2021.

[59] An G, Fitzpatrick BG, Christley S, Federico P, Kanarek A, Neilan RM (2017). Optimization and control of agent-based models in biology: a perspective. Bull Math Biol.

[60] Pappalardo F, Fichera E, Paparone N, Lombardo A, Pennisi M, Russo G (2016). A computational model to predict the immune system activation by citrus-derived vaccine adjuvants. Bioinformatics.

[61] Russo G, Pennisi M, Viceconti M, Pappalardo F. In Silico Trial to test COVID-19 candidate vaccines: a case study with UISS platform. 2020. 10.1186/s12859-020-03872-0 PMC7733700 33308153

[62] Pennisi M, Russo G, Sgroi G, Bonaccorso A, Parasiliti Palumbo GA, Fichera E (2019). Predicting the artificial immunity induced by RUTI® vaccine against tuberculosis using universal immune system simulator (UISS). BMC Bioinform.

[63] Pappalardo F, Russo G, Pennisi M, Parasiliti Palumbo GA, Sgroi G, Motta S (2020). The potential of computational modeling to predict disease course and treatment response in patients with relapsing multiple sclerosis. Cells.

[64] Bianca C, Brézin L. Modeling the antigen recognition by B-cell and T-cell receptors through thermostatted kinetic theory methods (2017).10.1142/S1793524517500723

